# Health insurance coverage and modern contraceptive use among sexually active women in Nigeria: Further analysis of 2018 Nigeria Demographic Health Survey

**DOI:** 10.1186/s40834-022-00187-8

**Published:** 2022-11-01

**Authors:** Obasanjo Afolabi Bolarinwa, Taiwo Oladapo Babalola, Oladayo Abayomi Adebayo, Kobi V. Ajayi

**Affiliations:** 1 Department of Global Public Health, Canterbury Christ Church University, Canterbury, UK; 2 Department of Public Health Medicine, School of Nursing and Public Health, University of KwaZulu-Natal, Durban, South Africa; 3 Institute of Governance, Humanities, and Social Sciences [PAUGHSS], PAN African University, Yaoundé, Cameroon; 4 R-DATS Consulting, Abuja, Nigeria; 5 Education, Direction, Empowerment, & Nurturing (EDEN) Foundation, Abuja, Nigeria; 6 Department of Health Behavior, School of Public Health, Texas A&M University, 77843 College Station, TX USA

**Keywords:** Health Insurance, Contraceptive methods, Family Planning, Sexually active women, Nigeria, DHS

## Abstract

**Background:**

Studies have shown that affordable health insurance can influence healthcare visits and increase the choice of medication uptake in sub-Saharan Africa. However, there is a need to document the influence of health insurance coverage and modern contraceptive use in order to encourage its uptake. Thus, this study examined the influence of health insurance coverage on modern contraceptive use among sexually active women in Nigeria.

**Methods:**

The secondary dataset utilised in this study were derived from the 2018 Nigeria Demographic and Health Survey (NDHS). Data analyses were restricted to 24,280 women of reproductive age 15–49 years who were sexually active in the survey dataset. Weighted bivariate and multivariable logistic regression models were used to examine the influence of health insurance coverage on modern contraceptive use while controlling for possible confounders. A Significant level of alpha was determined at p < 0.05 using STATA 16.0.

**Results:**

The prevalence of health insurance coverage and modern contraceptive use among sexually active women in Nigeria were 25.47% and 13.82%, respectively. About 1 out of every 4 sexually active women covered by health insurance were using a modern contraceptive, while 86.50% of the women not covered by health insurance were not using any modern contraceptive method. After adjusting for socio-demographic characteristics, the odds of using any modern contraceptive were significantly higher for sexually active women who were covered by any health insurance [aOR = 1.28; 95% (CI = 1.01–1.62)] compared to sexually active women not covered by health insurance in Nigeria.

**Conclusion:**

The study demonstrated that health insurance coverage is a significant driver of health service utilization, including modern contraceptive use. Health insurance benefits are recommended to be expanded to cover a broader spectrum of family planning services in Nigeria. More research is required to understand the influence of different health insurance schemes and the use of modern family planning methods in Nigeria.

## Introduction

Modern contraceptive use has been identified as one of the biggest public health achievements in the past century [1, 2]. It is currently recognized as a significant element in reaching environmental, economic, and socially sustainable development [3] because it may help improve child and maternal health outcomes [4, 5]. Contraceptives also prevent unwanted pregnancies and risky abortions, while condom use reduces sexually transmitted infections [6–8]. The United Nations Development Programme (UNDP) report estimated that out of 1.9 billion sexually active women worldwide, about 1.1 billion require contraception to limit or delay childbirth. The report further estimated that between the years 2000 and 2020, contraceptive use among sexually active women increased by 188 million globally; however, this increment is not homogenous across different regions [9, 10].

The most reliable form of contraceptive is the modern contraceptive [11], and its use has been on the increase in the past few decades [12]. For instance, modern contraceptive use is associated with increased inter-pregnancy interval and reduced mortality of under-five children [11]. Additionally, modern contraceptive use can prevent an estimated 60 million years of population-healthy life and 2.7 million infant deaths. Based on this usefulness, the global use of modern methods is increased to 76.80% in 2020 from 73.60% to 2000 [13]. Despite this increase in the worldwide modern contraceptives use, disparities exist among resource-constrained countries. For example, sub-Saharan Africa (SSA) countries have a lower prevalence of modern contraceptive use compared to developed nations, and this has been reported in Nigeria (13%) and many African nations [2, 14–16].

The underutilisation of modern contraceptives in Nigeria stems from a myriad of factors but mainly finds its root in existing health system failures such as weak health policy formulation and poor motivation among health care workers, which has led to a massive brain drain of medical workers, weak health infrastructures, and poor health sector financing [17]. As a result, government initiatives that support access to sexual and reproductive health commodities, including family planning, remain elusive and suboptimal despite a few programs, such as the Nigerian Urban Reproductive Health Initiative, funded by Bill and Melinda Gates Foundation. Moreover, the issue of access to family planning services, in particular continues to be a burgeoning problem in the country mainly due to funds [18]. The low prevalence of modern contraceptive use in the country is further compounded by a lack of universal health coverage (UHC), where the majority of Nigerians pay out-of-pocket for health expenditure and given that the country has an alarmingly high poverty rate, this does not present as a surprise [19].

Evidence has demonstrated that UHC is paramount to increased access to maternal health care utilization [20, 21]. One of the mandates of UHC is to ensure equal access to healthcare services irrespective of the geographical location or socioeconomic status [20]. For instance, the Abiye program piloted in one western state in Nigeria is an excellent example of how UHC can improve access and utilisation of modern contraception in the country [22]. Through the provision of free fully integrated maternal and child care facilities and referrals to all patients, the program reported a 69.6% increase in facility utilization, an increase of deliveries undertaken by skilled birth attendants from 43.3 to 69.6%, and an overall reduction of maternal mortality by 31% after two years of implementation in one site alone [4, 22]. However, due to the country’s health system challenges, there has been persistent inadequate national health insurance coverage in Nigeria compared to other developed countries [19]. For instance, since creating the National Health Insurance scheme in 1999, less than 5% of the overall population has health insurance coverage [19, 23]. While efforts to increase access to UHC in the country are underway and laudable, challenges still persist [24].

A 2018 report by health policy plus among seven low-and-middle income countries that committed to the global family planning 2020 initiative indicates that there is an association between health insurance cove rage and family planning use [25] and that most sexually active women have inadequate access to health insurance [34]. However, studies conducted in Nigeria have yet to examine the interconnectedness of modern contraceptive use and health insurance coverage among sexually active women [26–28]. Rather, they have focused on influencing factors, diffusion effects, the behavior of users, inequalities, and the unmet need for modern contraceptives. Considering the benefits of health insurance coverage in increasing maternal health care services and the low use of modern contraceptives in Nigeria, it is important to understand the nexus between health insurance coverage and modern contraceptive use.

Thus, this study examines the relationship between health insurance coverage and modern contraceptive use among sexually active women in Nigeria using the most recent Nigeria Demographic Health Survey (NDHS). The research outcome is envisaged to help policymakers know if investment in health insurance could increase modern contraceptive use and enable stakeholders to make informed policy decisions that will increase modern contraceptives in Nigeria.

## Data and methods

### Design and setting of the study

We used data from the most recent Nigeria Demographic Health Survey (NDHS), which was conducted in 2018, to examine the influence of health insurance coverage on modern contraceptive use among sexually active women in Nigeria [29]. The 2018 NDHS consists of a nationally representative sample of 41,821 women of reproductive age 15–49 years. The survey adopted a stratified two-stage sampling technique to provide valid and reliable estimates on key development indicators at national and sub-national levels. The NDHS captures data on modern contraceptive usage and health insurance coverage. The survey also captures information on respondents’ socio-demographic characteristics, which can be used to control the influence of health insurance coverage on modern contraceptive use among the target population in our study [29].

### Sample characteristics

The NDHS dataset considers sexually active women as those who engaged in sexual intercourse within 30 days prior to the survey. Out of 41,821 women between the age of 15–49 years covered in the 2018 NDHS, a weighted sample of 24,280 women met the definition of sexually active women. Since our focus in this study is on sexually active women in Nigeria, the study sample size is restricted to the 24,280 sexually active women in the dataset.

### Outcome variable

The dependent variable in this study is modern contraceptive use. Women were asked if they or their partners are ‘currently doing something or using any method to delay or avoid getting pregnant?’ Those who responded in the affirmative were asked to mention the type(s) of contraceptive methods currently in use which could either be any modern methods (i.e., pill, intrauterine device (IUD), injections, male condom, female sterilization, implants/Norplant, lactational amenorrhea, female condom, emergency contraception, standard days method and/or other modern methods) or traditional methods (Period abstinence, withdrawal and/or other traditional methods). In this study, the survey responses to these questions were dichotomized as 1 = currently using any modern method and 0 = not currently using any method or currently using any traditional method. This categorization of modern contraceptive use as the dependent variable aligns with previous related studies [3, 26, 30].

### Explanatory variables

The first explanatory variable used in this study was health insurance coverage and was used as a key independent variable, whilst the second domain of explanatory variable was the socio-demographic characteristics of the respondents, which were used as the covariates.

#### Key independent variable

In this study, the independent variable was health insurance coverage selected based on its association with maternal health service utilisation in previous studies [31, 32]. The study participants responded to the question, ‘Are you covered by any health insurance?’ We coded the health insurance coverage as “0 = no” (reference category = RC) if the respondent is not covered by health insurance, whilst respondent is covered by health insurance was coded as “1 = yes”.

#### Covariates

We identified the covariates based on similar studies on family planning [26, 30, 33, 34] and the availability of the variables in the 2018 NDHS dataset. The covariates constitute the second explanatory domain and are included in the analysis to control for possible confounders in the study analytical model. These include the main socio-demographic characteristics of survey respondents: age group, level of education, marital status, ethnicity, religion, employment status, wealth index, place of residence, geo-political regions, media, parity level, and desire for more children. The available media channels included and measured separately in the study are watching television, listening to the radio, and reading newspapers or magazines [29]. In the DHS, Principal Component Analysis (PCA) was used to generate a wealth index from a range of information collected on household assets, including type and volume of consumer goods owned, means of transportation, housing features, and ownership of animals [29].

### Data analysis

The frequency distribution and cross-tabulation of sexually active women were first described by variables on health insurance coverage, socio-demographic characteristics, and modern contraceptives. This was followed by examining the associations between modern contraceptive use and each explanatory variable, such as health insurance coverage and socio-demographic characteristics, by using chi-square (*X*^2^). Logistic regression models were used to examine unadjusted and adjusted odds ratios (AOR) of the effects of respondents’ health insurance coverage and their socio-demographic characteristics on uptake of modern contraceptive methods at 95% confidence intervals (CIs). All frequency distribution analyses were weighted using recommended DHS sampling weight of “v005/1000000” to account for the complex survey sampling design of DHS and ensure representativeness of the model estimates at the national level. All data were analyzed using STATA, version 16.0 and a significant alpha level was determined at p < 0.05.

### Ethical approval

Since the authors of this manuscript did not collect the data, we sought permission from the MEASURE DHS website, and access to the data was provided after our intent for the request was assessed and approved on the 10th of January 2021. The DHS surveys are ethically accepted by the ORC Macro Inc. Ethics Committee and the Ethics Boards of partner organizations in different countries, such as the Ministries of Health. The women who were interviewed gave either written or verbal consent during each of the surveys.

## Results

Table 1 presents results on the frequency distribution, percentage and cross-tabulation of women covered by health insurance, selected socio-demographic characteristics, and modern contraceptive usage among sexually active women in Nigeria. The prevalence of modern contraceptive use among sexually active women in Nigeria was 13.82%. Health insurance coverage shows that 25.47% of sexually active women in Nigeria covered by health insurance were using modern contraceptives, while 86.50% of those not covered by health insurance were not using modern contraceptives. Sexually active women with no desire for more children (21.26%) were currently using modern contraceptives, and 95.35% of sexually active women without educational backgrounds were not using modern contraceptives in Nigeria. All included explanatory variables were significantly associated with modern contraceptive use among sexually active women at p < 0.001.

**Table 1:** Weighted frequency and percentage distribution of health insurance coverage, selected socio-demographic characteristics, and modern contraceptive use among sexually active women in Nigeria

Variables N = 24,280	Modern contraceptive use				
**Health Insurance Coverage**	**Frequency (n)**	**Percentage (%)**	**No (%)**	**Yes (%)**	**X** ^**2**^ **p-value**
No	23,648	97.39	86.50	13.50	p < 0.001
Yes	632	2.61	74.53	25.47	
**Age group**					p < 0.001
15–24	5,868	24.17	91.08	8.92	
25–34	9,598	39.53	84.84	15.16	
35 +	8,814	36.30	84.39	15.61	
**Level of education**					p < 0.001
No education	10,872	44.78	95.35	4.65	
Primary	3,466	14.27	83.60	16.40	
Secondary and above	9.942	40.95	77.06	22.94	
**Marital status**					p < 0.001
Single	1,212	4.99	72.34	27.66	
Currently Married	22,103	91.03	87.26	12.74	
Cohabitation	666	2.74	81.92	18.08	
Previously married	299	1.23	72.67	27.33	
**Currently working**					p < 0.001
No	7,355	30.29	91.22	8.78	
Yes	16,925	69.71	83.99	16.01	
**Parity**					p < 0.001
No child	2,760	11.37	89.35	10.65	
1–3	9,941	40.94	86.08	13.92	
4 and above	11,579	47.69	85.52	14.48	
**Desire for more children**					p < 0.001
Want more	17,140	70.59	88.99	11.01	
Undecided	1,435	5.91	82.27	17.73	
No desire	5,705	23.50	78.74	21.26	
**Ethnicity**					p < 0.001
Hausa	10,647	43.85	93.90	6.09	
Yoruba	3,243	13.36	71.78	28.22	
Igbo	2,790	11.49	80.58	19.42	
Others	7,600	31.30	83.57	16.43	
**Religious**					p < 0.001
Christianity	9,395	38.69	77.41	22.59	
Islam	14,765	60.81	91.69	8.31	
Traditional & Others	120	0.49	95.04	4.96	
**Read Newspaper**					p < 0.001
No	21,140	87.07	87.76	12.24	
Yes	3,140	12.93	75.61	24.39	
**Listening to radio**					p < 0.001
No	11,176	46.03	90.58	9.42	
Yes	13,104	53.97	82.44	17.56	
**Watch television**					p < 0.001
No	12,960	53.38	92.91	7.09	
Yes	11,320	46.62	78.48	21.52	
**Wealth Index**					p < 0.001
Poorest	5,047	20.79	96.02	3.98	
Poorer	5,190	21.38	92.61	7.39	
Middle	4,541	18.70	87.13	12.87	
Richer	4,672	19.24	78.84	21.16	
Richest	4,830	19.89	75.22	24.78	
**Place of residence**					p < 0.001
Urban	9,751	40.16	79.07	20.93	
Rural	14,529	59.84	90.96	9.04	
**Region**					p < 0.001
North-Central	3,097	12.76	82.75	17.25	
North-East	4,345	17.89	91.17	8.83	
North-West	8,612	35.47	93.04	6.96	
South-West	1,978	8.15	83.43	16.57	
South-South	2,490	10.26	79.61	20.39	
South-West	3,758	15.48	73.36	26.64	
Modern contraceptive use	24,280	100%	86.18	13.82	

Weighted NDHS (2018)

Table 2 shows the results of health insurance coverage selected socio-demographic characteristics modern contraceptive use in Nigeria. There is a significant association between the key independent variable (Health insurance coverage) and modern contraceptives among sexually active women in Nigeria. The adjusted odds ratio (AOR) shows that Nigerian sexually active women who were covered by health insurance [aOR = 1.28; 95%(CI = 1.01–1.62)] had higher odds of using modern contraceptives compared to sexually active women who had no health insurance coverage in Nigeria.

In the same vein, the results of the socio-demographic characteristics and modern contraceptive use among sexually active women in Nigeria show that age of respondents, parity, level of education, marital status, children ever born (parity), desire for more children, ethnicity, religion, watching television, wealth index, place of residence, and region of residence were significantly associated with modern contraceptive use among sexually active women in Nigeria.

Higher odds ratio significant results show that those with higher education [aOR = 2.69; 95%(CI = 2.22–3.27)], women who were affiliated with the Yoruba ethnic group [aOR = 1.86; 95%(CI = 1.34–2.59)], women who watch television [aOR = 1.22; 95%(CI = 1.05–1.42)], and those within the richest wealth index [aOR = 2.33; 95%(CI = 1.80–3.01)] had higher odds of using modern contraceptive compared to those who had no educational background, women affiliated with Hausa ethnic group, those who do not watch television and women within poorest wealth index. On the other hand, sexually active women in the older age group of 35 years and above [aOR = 0.73; 95%(CI = 0.60–0.90)], those cohabiting [aOR = 0.24; 95%(CI = 0.16–0.36)], women residing in the rural area [aOR = 0.79; 95%(CI = 0.70–0.90)], and those residing in the South-East [aOR = 0.45; 95%(CI = 0.33–0.62)] had lower odds of using modern contraceptive in Nigeria compared to women who were within the younger age group (15–24), those that were single, women residing in the urban area, and those residing in the North-Central region of the country.

**Table 2:** Results of bivariate and multivariate logistics regression of health insurance coverage, selected socio-demographic variables on modern contraceptive use among sexually active women in Nigeria

Variables N = 24,280	Modern contraceptive use	
	**Unadjusted**	**Adjusted**
**Health Insurance Coverage**	**cOR [95% CI]**	**aOR [95% CI]**
No	RC	RC
Yes	2.19***[1.73–2.77]	1.28*[1.01–1.62]
**Age group**		
15–24	RC	RC
25–34	1.82***[1.61–2.07]	1.07[0.92–1.25]
35 +	1.89***[1.63–2.18]	0.73**[0.60–0.90]
**Level of education**		
No education	RC	RC
Primary	4.03***[3.42–4.74]	2.17***[1.82–2.59]
Secondary and above	6.11***[5.24–7.12]	2.69***[2.22–3.27]
**Marital status**		
Single	RC	RC
Currently Married	0.38***[0.31–0.48]	0.25***0.18–0.34]
Cohabitation	0.58**[0.42–0.79]	0.24***[0.16–0.36]
Previously married	0.98 [0.70–1.38]	0.38***[0.25–0.59]
**Currently working**		
No	RC	RC
Yes	1.98***[1.75–2.24]	1.10[0.95–1.26]
**Parity**		
No children	RC	RC
1–3	1.36**[1.13–1.63]	3.09***[2.47–3.87]
4 and above	1.42***[1.19–1.69]	5.00***[3.81–6.57]
**Desire for more children**		
Want more	RC	RC
Undecided	1.74***[1.38–2.20]	1.53**[1.19–1.97]
No desire	2.18***[1.97–2.42]	1.81***[1.58–2.06]
**Ethnicity**		
Hausa	RC	RC
Yoruba	6.06***[4.85–7.57]	1.86***[1.34–2.59]
Igbo	3.71***[2.93–4.71]	1.25[0.82–1.91]
Others	3.03***[2.44–3.76]	1.33[0.97–1.81]
**Religious**		
Christianity	RC	RC
Islam	0.31***[0.27–0.35]	0.55***[0.44–0.70]
Traditional & Others	0.18***[0.08–0.39]	0.28**[0.14–0.57]
**Read Newspaper**		
No	RC	RC
Yes	2.31***[2.02–2.64]	1.06[0.92–1.22]
**Listening to radio**		
No	RC	RC
Yes	2.05***[1.82–2.30]	1.03[0.90–1.17]
**Watch television**		
No	RC	RC
Yes	3.59***[3.16–4.09]	1.22**[1.05–1.42]
**Wealth Index**		
Poorest	RC	RC
Poorer	1.92***[1.46–2.52]	1.42**[1.10–1.84]
Middle	3.56***[2.85–4.44]	1.76***[1.40–2.22]
Richer	6.47***[5.21–8.03]	2.34***[1.82–3.01]
Richest	7.94***[6.41–9.83]	2.33***[1.80–3.01]
**Place of residence**		
Urban	RC	RC
Rural	0.37***[0.33–0.43]	0.79***[0.70–0.90]
**Region**		
North-Central	RC	RC
North-East	0.46***[0.38–0.56]	1.05[0.87–1.26]
North-West	0.36***[0.28–0.46]	0.99[0.78–1.26]
South-East	0.95[0.79–1.15]	0.45***0.33–0.62]
South-South	1.23 [1.04–1.44]	0.60***[0.50–0.72]
South-West	1.74***[1.48–2.04]	0.74**[0.60–0.92]

Weighted NDHS, 2018

RC = Reference category; CI = confidence interval; cOR = unadjusted odds ratios; aOR = adjusted odds ratios

* p < 0.05; ** p < 0.01; *** p < 0.001

## Discussion

This study examined the relationship between health insurance coverage and modern contraceptive use among sexually active women in Nigeria. Our results showed an overall low modern contraceptive use prevalence of 13.82%. Previous studies have found a comparable prevalence rate of modern contraceptive use in Nigeria, ranging from 12.60 to 18.70% [35, 36]. However, our result is lower than that of Ghana (21.00%) and Guinea (74.37%) but within the high percentile in SSA (17%) [37, 38]. In all, these results suggest that family planning service utilization is low in Nigeria. Therefore, our results show the need for effective public health interventions to increase modern contraceptive use in Nigeria.

Among sexually active women, only a small proportion (25.47%) with health insurance utilized a modern contraceptive. Although the result is higher than the 8.50% health insurance rate of women in SSA, Nigeria ranks 16 out of 24 SSA countries, with a prevalence rate of 2.80% vs. 62.40% in Ghana [23]. Still, Nigeria currently has significantly lower levels of health insurance access compare to Ghana (40.00%), Kenya (20.00%), and Ethiopia (14.00%) [25].

Several factors impact the health insurance scheme in Nigeria. First, as noted earlier, inadequate financing in the country’s health care sector continues to pose a significant barrier to health insurance access [39]. Furthermore, considering that the health insurance scheme is largely financed by the NHIS and covers only the formal sector, demands surpass available funds, thus leaving a huge number of the working population without access to health insurance coverage [25, 39]. Also, the health care system in Nigeria is heavily fragmented. These issues make it challenging to coordinate and distribute resources across the levels and hierarchy [23, 39]. Given these concerns, it is not surprising we found that an approximated 8 out of every 10 sexually active women do not have access to health insurance, resulting in sub-optimal contraceptive use as seen in previous studies [38, 39].

The relationship between health insurance coverage and contraceptive use in our study supports the theory that health insurance access is a significant predictor of health service utilization, such as preventive health screening and overall health outcomes [25]. Our analysis revealed that sexually active women in Nigeria with health insurance coverage were more likely to use modern contraceptives compared to their counterparts with no health insurance coverage. This is possible because access to health insurance coverage could encourage healthcare utilisation which could encourage the uptake of modern contraceptive in return [31]. The lack of health insurance coverage may explain the low levels of modern contraceptive use. Although health insurance coverage is a significant predictor of family planning services, evidence suggests that health insurance alone may not improve contraceptive use. Indeed, the knowledge of and willingness to utilize family planning services may also be barriers to contraceptive uptake [25, 40]. Other challenges from previous studies associated with using family planning services include poor supply chain, structural inequality and provider-patient poor communication [25].

This study also showed a significant association between some of the socio-demographic variables included in this study and modern contraceptive use. We found that educational level, parity, desire to have more children, watching television, ethnicity, and wealth index were significant predictors of modern contraceptives use. These findings, though significant, are mixed compared to previous studies. For example, findings from researchers in Ethiopia, Guinea, and Nigeria, support our results as they found higher odds of modern contraceptive use in people with secondary and tertiary education than in people without formal education [36, 38]. Similarly, our results align with Seidu et al., [38] findings suggesting that exposure to media, such as watching television, increases the likelihood of contraceptive use [27]. However, relating to the wealth index, our finding is contrary to a study conducted by Aviisah et al., [15]. We found that chances of utilizing contraceptives increased as women moved from poorest to richest. These inconsistencies notwithstanding, income level, which is a significant predictor of wealth by default influences health behaviors [35]. This is also in line with a study conducted by Seidu et al., [38] which suggests that rich women may have high health literacy, can partake in major family decisions, and are more knowledgeable of the benefits of family planning than women from poor households who may otherwise have low educational levels with poor knowledge of family planning services [38].

Furthermore, we found that marital status, area of residency, religion, region, and age, were associated with lower odds of contraceptive use. These findings are consistent with previous studies [17, 39]. For instance, these studies showed that the likelihood of modern contraceptive use was higher among younger-aged women (15–19 years) than their older-aged counterparts (45–49 years). Our results also suggest that several multifaceted factors may affect family planning services. For example, it is plausible that older-aged women (e.g., 35 years and above) may be menopausal as such chances of conception are unlikely than younger-aged women with higher chances of conception. In the same vein, those who are previously married may be of advanced age than single women; thus, family planning use might be lower among these subgroups [36]. A consistent and important finding is the rural-urban variations in modern contraceptive use. It appears that women in rural areas have lower levels of education, low income, and may have less decision-making powers which may negatively affect their contraceptive use [34, 38]. Rural areas may also have poorly equipped healthcare centers and providers, which may explain our results [41]. These findings suggest that a holistic approach to improving modern contraceptive use is important [25].

### Strengths and limitations

There are several strengths to our study. The results of this study can inform public health efforts and policy. Secondly, we applied the NDHS weighting strategy to obtain precise population estimates, making our inferences generalizable and replicable. Lastly, to the best of our knowledge, our study is the first to analyze the health insurance coverage on contraceptive use among sexually active women vs. the general female population. Although our analysis is timely and contributes significantly to the literature, there are noteworthy limitations. Firstly, our outcome variable, modern contraceptives, is a combined variable of all types of contraceptives (e.g., injectables, condoms, or IUD); thus, our results may not show the true differentials among which types of contraceptives are preferred or more utilized and why. This may also impact knowledge about which of the contraceptive types are covered by health insurance. Secondly, our key independent variable was dichotomized; hence we did not analyze if the women in our sample had a public or private insurer. Information about insurance types may be important to provide an in-depth understanding of the health insurance scheme in Nigeria related to family planning services. Another inherent limitation of our study is that we utilized a cross-sectional design, which is self-reported and prone to misclassification and recall bias. We did not include other variables (e.g., provider’s perspective) that may explain our results. In the same vein, the survey was conducted 4 years ago, and the coverage of insurance and modern contraceptive use may have improved. Lastly, service availability and accessibility variables are important in explaining the outcome variable of the study but are not included in the NDHS. However, the DHS is a highly cited medical dataset used to draw inferences. Despite these limitations, our findings are comparable with previous studies suggesting that the underlying factors impacting family planning services are pervasive and consistent.

### Policy implications

This study has important implications for policy, public health professionals, and researchers. The relationship between health insurance and modern contraceptive use demonstrated in this study suggests that lack of health insurance access is a significant barrier to utilizing family planning services in Nigeria. Stakeholders should consider expanding health insurance benefits to cover more choices of family planning services. Policymakers should also consider continuous and targeted health education messages to dispel misinformation or myths held about family planning services [42].

## Conclusions and recommendations

Our study examined the relationship between health insurance and modern contraceptive use among sexually active women. The study concluded that health insurance coverage influenced modern contraceptive use among sexually active women in Nigeria. The study further showed that selected socio-demographic variables such as educational level, parity, desire to have more children, watching television, ethnicity, and wealth index were significantly associated with modern contraceptive use among sexually active women in Nigeria. We recommend that subscription to health insurance coverage should be made affordable or free in Nigeria to attract subscriptions from sexually active women within low-socioeconomic status such as lower education and poorest wealth index.

Future research is needed to understand which contraceptive type is most preferred among sexually active women who subscribed to health insurance coverage in Nigeria.

## Data Availability

The DHS dataset is freely available for use upon request at https://dhsprogram.com/data/available-datasets.cfm. After the request has been approved, a de-identified dataset will be made available.

## Declarations

### Authors’ details

OAB: ^1^Department of Global Public Health, Canterbury Christ Church University, Canterbury, United Kingdom; ^2^Department of Public Health Medicine, School of Nursing and Public Health, University of KwaZulu-Natal, Durban, South Africa. bolarinwaobasanjo@gmail.com. TOB:^3^Institute of Governance, Humanities, and Social Sciences [PAUGHSS], PAN African University, Yaoundé, Cameroon. babalolataiwoo@gmail.com. OAA:^4^R-DATS Consulting, Abuja, Nigeria. dayoadebayo@r-datsconsulting.org. KVA:^5^Education, Direction, Empowerment, & Nurturing (EDEN) Foundation, Abuja Nigeria; ^6^Department of Health and Kinesiology, Texas A&M University, College Station, TX, 77843, USA. kobiajayi@gmail.com.

## Abbreviations

NDHS: Nigeria Demographic and Health Survey
UNDP: United Nations Population Division
UN DESA: United Nations Department of Economic and Social Affairs
NHIS: National Health Insurance Scheme of Nigeria
PCA: Principal Component Analysis
IUD: Intrauterine device
SSA: sub-Saharan Africa
DHS: Demographic health survey
